# “In small places, close to home”: Urban environmental impacts on child rights across four global cities

**DOI:** 10.1016/j.healthplace.2023.103081

**Published:** 2023-07-26

**Authors:** Emily Gemmell, Dina Adjei-Boadi, Asesh Sarkar, Niloofar Shoari, Katherine White, Svetlana Zdero, Hallah Kassem, Tina Pujara, Michael Brauer

**Affiliations:** a School of Population and Public Health, University of British Columbia, Vancouver, 2206 West Mall, Vancouver, BC, V6T 1Z4, Canada; b Department of Geography and Resource Development, University of Ghana, MR28+9MQ, Doutor J.B. Danquah Avenue, Accra, Ghana; c Department of Architecture and Planning, Indian Institute of Technology, Haridwar Highway, Roorkee, Uttarakhand, 247667, India; d MRC Centre for Environment & Health, Department of Epidemiology and Biostatistics, Imperial College London, St Mary’s Campus, Norfolk Place, London, W2 1PG, United Kingdom; e Institute for Health Metrics and Evaluation, Population Health Building, Hans Rosling Center, 3980 15th Ave. NE, Seattle, WA, 98195, USA

**Keywords:** Child rights, Urban health, Child health, Child development, Healthy cities, Urban planning

## Abstract

Urban environments influence child behaviours, exposures and experiences and may affect health, development, achievement and realization of fundamental human rights. We examined the status of eleven UN Convention on the Rights of the Child articles, in a multi-case study across four global cities. Within all study cities, children experienced unequal exposure to urban environmental risks and amenities. Many violations of child rights are related to car-based transportation systems and further challenged by pressures on urban systems from rapid population increases in the context of climate change. A child rights framework provides principles for a collective, multi-sectoral re-imagination of urban environments that support the human rights of all citizens.

## 1 Background

“Where, after all, do universal human rights begin? In small places, close to home […] Unless these rights have meaning there, they have little meaning anywhere.”(Roosevelt, 1999)

The drafters of the United Nations Universal Declaration of Human Rights emphasized everyday environments as the primary settings where human rights are actualized. Most profoundly experienced in the “small places,” human rights also have little meaning unless equally applied to all people, including the smallest (O’Neill, 2005). Child rights in urban environments matter, not only because of the powerful impacts of early exposures on health and development and because children are the future workforce, innovators and leaders of societies but, from a justice and equity standpoint, because children are human beings. The UN Convention on the Rights of the Child (UNCRC), as a conceptual and legal framework, reflects shared human values across a range of historical, geo-political, cultural and religious contexts (UN General Assembly, 1989; Freeman, 2020). As the world’s most widely-ratified international treaty, the UNCRC outlines children’s fundamental human rights in 54 articles, with 41 substantive articles addressing nearly every aspect of child life and experience (UN General Assembly, 1989). The UNCRC is intended to guide policy and legislation in member States, with progress monitored through State reporting to the Committee on the Rights of the Child. However, consideration of child human rights in policies related to urban environments is often inconsistent (Child and Youth Law Section Canadian Bar Association, 2020; Canadian Bar Association, 2022). Efforts to ensure child rights in urban contexts have been taken by an increasing number of municipal and local governments, and frameworks developed to support subnational efforts (UNICEF, 2022; Vakerelska et al., 2022). However, a holistic understanding of how modern urban physical environments influence child rights in and across cities is lacking; assessments are often countrywide (Payne, 2017; Human Rights Council, 2022), or limited to specific projects (Wridt et al., 2015), issues (Human Rights Council, 2017) or neighbourhoods (Rakhimova et al., 2022). The dynamic, complex interactions between actors, agencies, markets and populations that shape cities may also obscure the question of who is responsible for upholding specific child rights related to these environments (O’Neill, 2005). In this paper, we examined the ways in which children’s human rights described in eleven UNCRC articles are supported or undermined by urban built environments and identify entities, actors and instruments responsible for actualization of these rights across four diverse urban contexts.

By 2050, more than two-thirds of the world’s children will live in cities, their experiences and exposures shaped by the form and quality of urban space (United Nations Department of Economic and Social Affairs Population Division, 2019). Since adoption of the UNCRC, rapid rural-urban migration, expansion of cities, social, geo-political and climate changes have altered the environments in which children’s rights are experienced, with potential impacts on multiple child health and developmental outcomes (Ye et al., 2022). In addition, children’s experiences and exposures may vary dramatically within cities due to unequal access to, distribution and quality of urban amenities (Gemmell et al., 2022). To gain a broad understanding of how urban environments currently influence the realization of children’s human rights within and across the study cities, we addressed the following questions using single and multi-case methods:

1. How do characteristics of urban environments support or undermine the realization of children’s rights?
2. What entities, actors and instruments are responsible to ensure actualization of these rights?

## 2 Methods

We conducted a multiple-case study across four diverse urban contexts: Accra, Ghana; Delhi, India; London, UK and Vancouver, Canada (Creswell and Poth, 2018; Yin, 2018). Study cities were selected from countries that have ratified the UNCRC treaty and vary in geography, culture, density, socioeconomic development and demographics, and for which at least one co-author had in-depth expertise on local urban environments. For this research, a child was considered to be a person between birth and 18 years of age, and “urban environments” include major physical characteristics of cities. Though referred to as “cities”, the study areas are adjacent administrative areas that form continuous urban regions (Table 1).

We identified eleven UNCRC articles with evidence for direct impacts from urban environmental characteristics (Table 1). We based our search strategy for the study cities around these eleven UNCRC articles, focusing on specific environmental features or exposures but also allowing for flexibility and emergence of city-specific urban environmental factors with impacts on child rights (Table 1).

We searched for city-specific documentary evidence for how urban environments influence child rights and the duty-holders with explicit or implicit responsibility for ensuring the realization of these rights. Data collection for each city was led by a co-author with expertise in child health, environmental health, urban planning, architecture, and/or epidemiology within the local context. Data sources included peer-reviewed journal databases, international, federal, state/provincial, municipal and administrative area-level evaluations, reports and policy documents; non-governmental, community organization evaluations and reports; not-for-profit and media reports. Basic demographic data was collected for each city from existing open data sources (Office of the Registrar General and Census Commissioner, 2011; Ghana Statistical Service, 2021; Nomis Office for National Statistics, 2021; Government of India; Statistics Canada, 2022a).

Co-authors from each city examined documentary data within the framework of the research questions (Yin, 2018), identifying major themes, duty-holders and their domains of actions. Co-authors drew on local knowledge of formal and informal systems of influence, control, development and regulation, considering each case holistically, rather than focusing narrowly on a set of variables. We undertook this analytic approach as best suited to addressing the research questions within and across diverse, complex urban systems (Byrne, 2012). All evidence was then independently examined by the primary author to validate findings, with discrepancies resolved through discussion with co-authors. Co-authors provided high-level descriptive evidence summaries for each city, including major issues, barriers and facilitators to realization of child rights. We then conducted cross-case analysis, organizing single-case data for each city within a matrix to examine patterns across cities (Yin, 2018). We identified convergence and divergence in themes and summarized cross-case insights for urban environmental characteristic examined. Based on findings from this cross-case analysis, we developed a higher-level conceptual model (Yin, 2018) for how a child rights framework may inform priorities and action across all levels of society for healthy and just cities.

## 3 Results

**Fig. 1:**
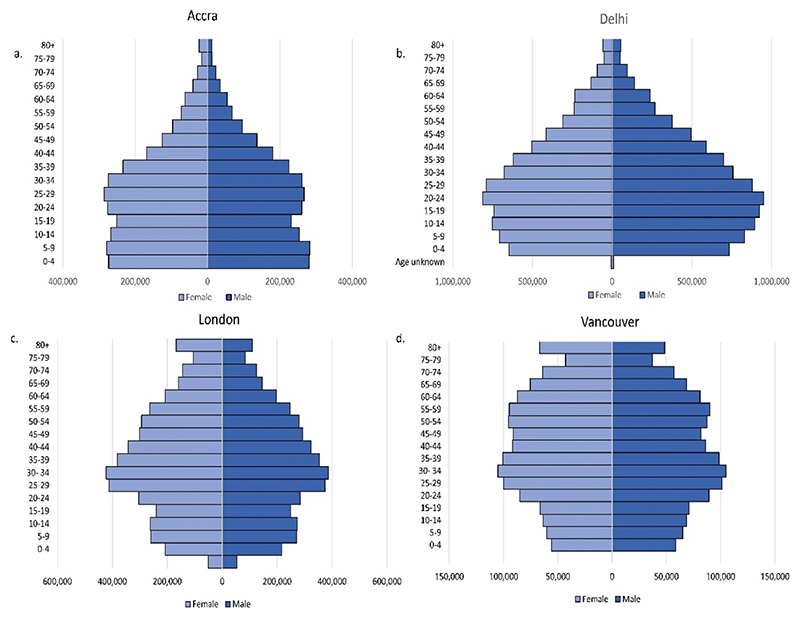
Population age structures for a. Accra (Ghana Statistical Service, 2021), b. Delhi* (Office of the Registrar General and Census Commissioner, 2011), c. London (Nomis Office for National Statistics, 2021) and d. Vancouver (Statistics Canada, 2022a), by gender and five-year age groups. *Delhi age/sex structure based on 2011 census data as 2021 census was delayed due to Covid-19.

The selected study cities are diverse in geography, history, climate, demographics, population density, culture and urban form and these factors uniquely shape local environments in which children’s rights are experienced (Table 2). The proportion of children within each city varies, with the highest proportion of children in Accra, and lowest in Vancouver (Fig. 1).

Below we provide brief, descriptive summaries of the status of children’s rights related to urban environments for each case city, followed by presentation of cross-case results (Table 3).

### 3.1 Accra, Ghana

On February 5, 1990, one week following the signing of the UN Convention on the Rights of the Child, Ghana became the first country in the world to ratify the treaty, committing to adopt its principles into national law (United Nations, 2022). Ghana has a rich history of leadership on child rights; playwright, author and child rights advocate Efua Sutherland celebrated the importance of play in “Playtime in Africa”, donated land for a public children’s park and inspired the work of the Mmofra Foundation, a non-profit supporting children’s rights to culture, the arts and public space (Sutherland and Willis E, 1962; Addo-Atuah, 2018). Though healthy urban policies exist, implementation is challenging in Accra’s complex policy context (Audia et al., 2021). Rapid urbanization, in combination with long-standinginadequate management and development of city service infrastructure, has overwhelmed resources, administrative and governance capacity to meet growing demands for housing, water, sanitation, waste management and transportation. An estimated 80% of Accra households rely on sachets (machine-sealed plastic bags) of water for drinking (Moulds et al., 2022). The resulting plastic waste contributes to flooding risk by blocking gutters, putting children at risk of injury or infection during urban flood events, especially those living in informal settlements in flood-prone areas (Amoako and Inkoom, 2018). In Accra, children are exposed to annual PM_2.5_ air pollution levels averaging 7.2 times higher than World Health Organization (WHO) guidelines (5 μg/m^3^) (World Health Organization, 2021), with higher exposures among girls, likely due to household cooking with biofuels (Arku et al., 2015; Alli et al., 2021; World Health Organization, 2021). Nearly all children are exposed to road traffic noise above WHO guidelines (World Health Organization, 2018), with higher exposures in low compared to high-income areas (Clark et al., 2021).

Among the urgent and competing demands on city resources, space and healthy urban design priorities such as provision of green spaces is neglected and the right to play is absent in policies related to children in Ghana (Adjei-Boadi et al., 2022). Widespread encroachment and rezoning of public spaces for other uses mean that many playground, parks and open spaces have disappeared. Those remaining are often unattractive and deserted due to lack of maintenance (Adjei-Boadi et al., 2022), while for-profit play spaces, though well-maintained, may be unaffordable for many families. Children play in informal open spaces such as school parks, undeveloped and unoccupied private lands, streets, pavements and large gutters. Informal spaces close to home provide opportunity for physical activity and socialization, but may expose children to traffic hazards, injury or infections (Adjei-Boadi et al., 2022).

Though exemplary participatory projects have been conducted by local non-profits, children’s views and opinions are not routinely considered on decisions regarding design and use of built environments (Adu-Gyamfi, 2013; Phillips and Serumaga-Musisi, 2020; Adjei-Boadi et al., 2022). Members of a youth advocacy assembly who organize and engage with local authorities felt that their input was not seriously considered.

*“… they take action only if they agree with us …”*

*“We were so passionate about the children’s park but we did not have the power to get it done.”*

(Adu-Gyamfi, 2013, p.1769)

### 3.2 Delhi, India

Children and young people make up approximately one-third of Delhi’s growing population, representing a major asset to the city’s social and economic future (Mody and Aiyar, 2011). However, many children live in informal settlements due to the city’s unmet housing needs in the face of rapid rural-urban migration (Manish, 2022). Often lacking city services, water and sanitation infrastructure, children may experience recurrent diarrhoea and parasitic infections, contributing to high rates of malnutrition, stunting and under-5 mortality (National Institute of Urban Affairs, 2020). Among the urban poor (two lowest quintiles of the National Family Health Survey Wealth Index), the under-5 mortality rate was nearly 60 per 1000 live births, compared to non-poor (<50/1000 live births) (National Institute of Urban Affairs, 2020). Inequality in access to water is reported, with the poorest households having least secure access to safe water (Kumar et al., 2021). Greenspace is inequitably distributed across the city. Of sixty-four wards in East and North East Delhi, with densities between 30,000–120,000 persons/km^2^, fourteen met the WHO criteria for per capita green space (9 m^2^), twenty-four had one or more playgrounds, while some lacked any parks at all (Mitchell et al., 2021). Gated communities may provide a strong sense of community, greenspace and low traffic that supports outdoor play (Bhonsle and Adane, 2016). However, heavy traffic and lack of pedestrian and cycling infrastructure pose serious risks outside these areas (Traffic Management Division, 2021), often limiting children’s active mobility to school and other destinations. Densely populated areas with higher proportions of children, fewer assets, lower electricity access and less greenspace have the highest urban heat risk (Mitchell et al., 2021). Though higher environmental risk is often linked to poverty, poor air quality violates the human rights of Delhi’s children across socioeconomic strata. Local PM_2.5_ levels may reach 400 μg/m^3^, 80 times the WHO guideline levels (Selokar et al., 2020; World Health Organization, 2021). High asthma rates and prevalence of acute respiratory infections (ARI) are seen in children across socio-economic strata (National Institute of Urban Affairs, 2020; Selokar et al., 2020). Various strategic plans at the national and city levels have been developed and initiated to improve air quality, however, the success of these programs to date has been mixed (Chatterji, 2020) and many are concerned that measures to mitigate harm may come too late for their children (Saxena, 2021). These issues are activating new social and political advocacy in India (Warrior Moms, 2023). India’s largest citizen-led urban movement is a sustained car-free initiative where cities across the country observe Sundays as car free days, closing certain roads, allowing them to be used by pedestrians and enjoyed as inclusive space for community activities (Dialogue and Development Commission of Delhi, 2022).

### 3.3 London, United Kingdom

Urban features that affect the rights of London’s children include air pollution, green space, traffic safety, affordable housing, neighbourhood violence, fast food outlet density, and alcohol and drug use (Greater London Authority, 2018a; The Greater London Authority, 2018; Mudway, 2019; Munro et al., 2022). Children from ethnic minorities, those living in poverty and those with disabilities are particularly susceptible to unequal exposures to these features, potentially contributing to health disparities over their lifespan.

The London Plan 2021, which establishes a 20-25-year development framework for the city, aims to ensure the provision of accessible and healthy spaces for children and young people. Though the plan recognizes a discretionary right to 10 m^2^ area of play space per child (The Greater London Authority, 2021), local authorities are not proactive in delivering the minimum required play space (Taylor and Nunes, 2022). Additionally, access to play spaces is inequitable across London schools, with those in central London often providing limited open and green spaces (Shoari et al., 2021). Roughly 800,000 (~60%) of London pupils attend schools with less than ten square meters per pupil of greenspace, of which 70% have no access to a public park in the immediate vicinity of their schools.

Breathing polluted air and childhood obesity are growing concerns in London that are directly influenced by urban features. Children are exposed to high NO_2_ (a marker for traffic-related air pollution) levels in school grounds, parks, and play spaces (Sheridan, 2019; Shoari et al., 2022). The issue has risen to prominence following the death of nine-year-old girl, Ella Kissi-Debrah, the first case of premature death officially recognized as due to unsafe levels of air pollution (Barlow, 2021). Despite this, air quality considerations are usually neglected in designing and planning of new developments (Taylor and Nunes, 2022). The prevalence of childhood obesity is unequally distributed across London and associated with deprivation. Barking and Dagenham, one of London’s most deprived local boroughs, has the highest prevalence of obesity among 11-year-olds while Richmond upon Thames, one of the least deprived, has the smallest figure (Office for Health Improvement & Disparities, 2019). London has considered several initiatives, especially in deprived neighbourhoods to address child obesity. The “School Superzone” initiative aims to create healthy spaces in 400 m buffers around schools by identifying school-specific problems and implementing corrective intervention, depending on local needs and circumstances (Fenton and Whiteman, 2021). In one deprived neighbourhood, a Superzone initiative engaged local businesses to support healthy eating, conducted surveys to inform traffic reduction, establish greenways and green links to schools from transit stops and engaged students and parents in an air quality campaign to raise awareness of this issue (Fenton and Whiteman, 2021).

The importance of involving children and young people in creating child-centric spaces from early stages in policy-making, design and development is also recognized in the London Plan 2021. Urban planners, architects, and local authorities are encouraged to understand how children and young people use spaces through participatory methods such as creative writing, photography and child-led walking tours, and to seek their views at every stage.(Mayor of London, 2019)

### 3.4 Vancouver, Canada

Throughout Metro Vancouver, issues related to urban environments for children are addressed in various municipal plans and some child-specific urban planning policies exist (City of Vancouver, 2015, 2020; Vancouver Board of Parks and Recreation, 2018; TransLink, 2022). However, many goals have yet to be realized equitably across neighbourhoods, and environmental, structural and social barriers remain. Most residents can access parks close to home, but overall greenness is higher in more affluent neighbourhoods (Quinton et al., 2022). Among Vancouver kindergarteners, the proportion of vegetation or paved surfaces around the child’s residence was associated with higher and lower scores, respectively, on measures of child development (Jarvis et al., 2022). Among 0–3-year-olds, higher greenspace and lower PM_2.5_ levels in residential neighbourhoods were associated with lower incidence of Attention Deficit Hyperactivity Disorder (ADHD) at 7 years, adjusting for neighbourhood-level marginalization (Yuchi et al., 2022). These recent findings suggest that environmental disparities may contribute to differences in developmental outcomes across Vancouver neighbourhoods.

Vancouver’s housing market is the most unaffordable in Canada (Statistics Canada, 2022b). High rents contribute to family stress, housing insecurity, parental time constraints, fewer resources and opportunities for children, especially those in racialized, Indigenous or lone-parent families (Gerlach et al., 2019; City of Vancouver, 2022). In a downtown urban neighbourhood with a high proportion of low-income residents and many Indigenous, immigrant, and visible minority families (Statistics Canada, 2016), social safety in the context of a city-wide opioid crisis, conflicting uses of outdoor space (e.g., play spaces being used as shelter by people experiencing homelessness) and lack of social cohesion and sense of community were identified as barriers to outdoor play and independent mobility (Gerlach et al., 2019). In higher-income neighbourhoods, fears of traffic and social danger have contributed to risk-averse parenting practices and social norms that limit children’s options for active leisure time and local social connections (Vlaar et al., 2019). Recent initiatives such as walking school bus, Play Streets and free public transit for children under 12 hold promise for improving children’s access to opportunity for social connection and physical activity (Porter et al., 2019; TransLink, 2022).

Opportunities for inclusion of child perspectives is present in local planning, a result of advocacy and collaboration between not-for-profit organizations and municipalities (Places for People, 2018). However, real safety concerns and limited freedoms due to risk-averse parenting norms mean that the reality for many children contrasts with official city plans (Gerlach et al., 2019). In the short term, prioritizing access to healthy outdoor environments near schools and childcare facilities would provide children with space for play and exposure to nature, which may be missing near homes in residential neighbourhoods. Longer-term actions include supporting the development of socially cohesive communities through housing policies that reduce residential instability, segregation, poverty, and inequality (Bakker and Dekker, 2012; Laurence, 2017).

### 3.5 Child rights across study cities

*“In all actions concerning children … the best interests of the child shall be a primary consideration”*

(UN General Assembly, 1989, p. 3)

In the complex processes that weigh multiple, competing demands for urban space and resources, the UNCRC provides a clear mandate to position the best interest of the child as a top priority (UN General Assembly, 1989, p. 2). Across contexts, various legislative, regulatory, planning and grassroots efforts to address urban air quality, traffic hazards, flood risk, noise and greenspace access have been undertaken and leaders uniformly affirm commitments to child well-being (Mayor of London, 2019; Dialogue and Development Commission of Delhi, 2022; Human Rights Council, 2022; City of North Vancouver, no date) However, changes are often elusive, slow or incomplete (Selokar et al., 2020; Issah, 2022; Shoari et al., 2022; Chew and Agbayani, 2023). The specific challenges to realization of child rights related to urban environments varied across study cities, with key differences related to economic development and governance capacity and structures. Accra and Delhi deal with complex challenges related to housing, water, sanitation, flooding and transportation infrastructure in the context of more limited resources, intense urban-rural migration and overlapping systems of control (Cobbinah and Erdiaw-Kwasie, 2016; Wells et al., 2019; Manish, 2022). In London and Vancouver, pressures on housing markets, harmful traffic-related exposures and social inequities result in dramatically unequal exposures to risks and opportunities among children (Gerlach et al., 2019; Shoari et al., 2021; Yuchi et al., 2022). Despite unique challenges and strengths across contexts, we identified common themes from cross-case analysis within the framework of the research questions.

1. Violations of child rights related to car-based transportation systems
2. Converging risks arising from inadequate services and infrastructure to meet the needs of rapid population increases in the context of climate change
3. Child rights are unequally experienced across socio-demographic groups
4. Collective action is required across society for equitable realization of child rights

Below we present cross-case findings for the child rights examined in this study, considering duty-holders and their domains of action, with evidence across cities summarized in Table 3.

### 3.6 Child rights and car-based transportation systems

Evidence from across cities shows that many of the most serious threats to children’s survival and development are linked to car-based transportation systems (Table 3). Vast tracts of public space are dedicated to car-based infrastructure, limiting land use for housing, public, green and natural space (Millard-Ball, 2022). Children are largely excluded from these public spaces due to traffic risks, which limit safe active travel options, local social interactions and access to child-relevant destinations (Vlaar et al., 2019; Shoari et al., 2023). Stressful or hazardous physical environments due to heavy traffic, noise, physical and social disorder or lack of greenspace influence child physical and mental health and development (Amram et al., 2011; Shoari et al., 2022) and may promote mistrust and isolation by limiting opportunities for local social interaction (Allport et al., 2019; Gerlach et al., 2019). Exposure to air pollution and noise is related to traffic density and proximity (Amram et al., 2011; Clark et al., 2021; Chauhan et al., 2023), with evidence for devastating impacts on child health and development (Barlow, 2021; Salvi et al., 2021). Measures to improve air quality and noise standards, speed limits, traffic law enforcement and pedestrian and cycling infrastructure hold potential to improve outcomes in current city contexts, however, these actions may be slow and incremental (Chatterji, 2020). The almost immediate improvements in air quality seen during Covid-19 lockdowns demonstrate the potential for radical change when all measures and resources are applied to meet a population health challenge (Dhaka et al., 2020). Our findings suggest that continued commitment to dominant car-based transportation systems in urban environments violates core human rights of children across study cities, with highest risks borne by children in lower income and marginalized groups (Table 3).

### 3.7 Child rights, urbanization and climate change

New challenges to children’s realization of their core human rights were seen across study cities, arising from imbalances between adequate infrastructure and services and increasing densification in the context of more frequent extreme weather events (Table 3). Intense pressures on housing stock and rapid development of land means that formal and informal green, play and public spaces may be appropriated for housing and transportation (Singh, 2018; Adjei-Boadi et al., 2022). Risks from car-based transportation infrastructure overlap with those from climate changes and increasing urbanization: higher populations and number of vehicles in cities have created hazardous air and noise levels (Amram et al., 2011; Selokar et al., 2020; Alli et al., 2021; Shoari et al., 2022), while more impervious surfaces increase flooding risk and may impact groundwater supply and water security (Amoako and Boamah, 2015; Biswas and Gangwar, 2021). Urban heat risk is higher with fewer trees, less greenspace and more impervious surfaces in dense areas (Mitchell et al., 2021), and existing housing materials may exacerbate heat risk (Wilby et al., 2021). Extreme heat and wildfire events, expected to increase in frequency, impact children’s opportunities for outdoor play, physical activity and social interaction, adding to existing barriers from traffic risks and lack of play and green space in urban areas (Allport et al., 2019; Shoari et al., 2021; Adjei-Boadi et al., 2022; Giordano, 2022). Converging risks from water insecurity, lack of sanitation and drainage infrastructure, inadequate greenspace, housing and lack of electricity in informal settlements mean that extreme weather events may pose life-threatening risks for many children in the most disadvantaged groups (Amoako and Inkoom, 2018; Mitchell et al., 2021; Tomar et al., 2021; Wilby et al., 2021). States, municipalities and local governments play a critical role in implementing policy, legal and regulatory frameworks, allocating resources and engaging international co-operation to uphold ratified rights for children within the State (Bartlett et al., 2021).

### 3.8 Unequal realization of child rights

Unequal exposure to risk or access to urban amenities impacting children’s rights was seen across cities. Dramatically different experiences and opportunities means that children’s human rights related to urban environments may be violated or upheld depending on their individual, family and community circumstances. Children’s rights are held without discrimination with regards to “*race, colour, sex, language, religion, political or other opinion, national, ethnic or social origin, property, disability, birth or other status*.” (UN General Assembly, 1989, p. 2) However, unequal access to healthy urban environments across socioeconomic, caste, ethnic origin, immigration or disability persist (Allport et al., 2019; Gerlach et al., 2019; Raushan et al., 2022; Chew and Agbayani, 2023; Odame et al., 2023) (Table 3). Urban systems that reduce biodiversity, deplete or harm the environment violate religious and spiritual traditions that teach respect and care for all living things (Kirmayer et al., 2000; Salmón, 2000; Francis, 2015; Bsoul et al., 2022) and may be a barrier to the “spiritual and moral development” (UN General Assembly, 1989, p. 8) of the child. Across the diverse Indigenous traditions within Canada, spirituality is “… defined not only in religious or spiritual terms but also in relation to the land” (Kirmayer et al., 2000, p. 612). Urban Indigenous children must be able to interact with natural space and ecosystems to fully realize the right to practice their own religion (UN General Assembly, 1989; Salmón, 2000). The right to enjoyment of *“… a full and decent life, in conditions which ensure dignity, promote self-reliance and facilitate the child’s active participation in the community”* (UN General Assembly, 1989, p. 7) includes children with disabilities. However, where evidence could be found across the study cities, it was clear that children living with disabilities were excluded from many public spaces and services (Sanderson et al., 2020; Chew and Agbayani, 2023; Odame et al., 2023). Though policies for inclusive public space and services exist, championing by private organizations, local communities and parents are often the force behind action to promote children’s dignified and active inclusion in everyday settings (Mensah et al., 2020; Special Needs Community, 2023).

### 3.9 A requirement for collective action across society

Ensuring children’s right to be heard (UN General Assembly, 1989, p. 4) in matters pertaining to the design of their neighbourhoods challenges the power relationships between children and adults in ways that the articles dealing with provision and protection do not. The “transformative potential of participation,” beyond the ideological value of respect for each citizen, is based on the real value that children’s voices contribute (Verhellen, 2015, p. 196). Often “strikingly direct and unvarnished”, honest and unafraid of controversy (Marris, 2019, p. 741), children are concerned with opportunities for play, social connection and access to nature, characteristics of a sustainable and healthy city for all ages (Gemmell et al., 2022). Children’s active presence and participation in neighbourhoods may catalyze social capital and sense of safety (Wood et al., 2013). Across contexts, local non-profit organizations (Places for People, 2018; Mayor of London, 2019; Mensah et al., 2020; Dialogue and Development Commission of Delhi, 2022), lead the way in elevating children’s voices on issues related to urban space. Despite these encouraging initiatives, there continue to be many decisions regarding urban environments made without consideration of child perspectives (Singh, 2018; Adjei-Boadi et al., 2022). Our findings indicate a need for involvement of children in the co-creation of urban space, beginning with relationship and routine, ongoing, inclusive and embedded participation in the decisions and environments of everyday life (Verhellen, 2015).

**Fig. 2:**
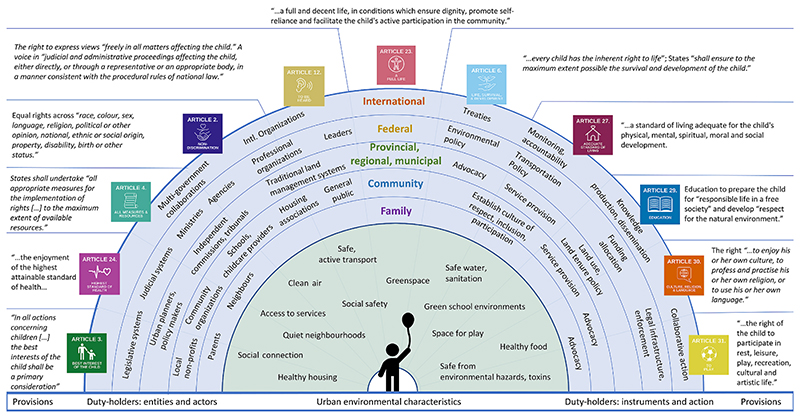
UN Convention on the Rights of the Child articles related to urban environments, and the duty-holders responsible for actualization of these rights in cities.

Across the widely diverse study cities, major categories of duty-holders responsible for upholding child rights related to urban environments and their domains of action were strikingly similar and are summarized in Fig. 2. At the most proximal level, grass-roots advocacy on the part of parents and local non-profits was essential promoting children’s rights to healthy urban space across contexts (Gill, 2015; Mensah et al., 2020; Dialogue and Development Commission of Delhi, 2022; Warrior Moms, 2023). Schools, childcare, social workers and health care workers and institutions are also important duty-holders, with deep knowledge of and responsibility for how child rights are being actualized in day-to-day environments within their domains of influence. The right to education, optimizing individual potential and preparing children for a “responsible life in a free society”, also encourages “the development of respect for the natural environment” (UN General Assembly, 1989, p. 9). Duty-holders within childcare and educational systems hold responsibility to advocate for child access to natural environments that support health, development, cognitive and behavioural outcomes (Kuo et al., 2019), instill a sense of respect, connection, and responsibility for local spaces and wildlife (Ärlemalm-Hagse’r, 2013) and mitigate disparities in home neighbourhood quality. Multiple agencies and actors across municipal, provincial and federal levels hold responsibility for complex and interacting systems, policies and infrastructure that impact child rights in the study cities (Cobbinah et al., 2020; Biswas and Gangwar, 2021; City of Vancouver, 2020; Munro et al., 2022). Given the evidence for unequal realization of rights for children in marginalized groups, awareness at each level of government is needed to prevent adoption of policies that (intentionally or unintentionally) systematically discriminate against groups (Nardone et al., 2020) and recognize the continued impacts of historical discrimination on the rights of urban children (Gerlach et al., 2019; Kumar et al., 2021; Odame et al., 2023).

City leaders and governments across the study cities were key players in articulating vision and providing direction to uphold child rights in local policies, land use, transportation planning and maintenance that shape everyday urban environments (Places for People, 2018; Mayor of London, 2019; TransLink, 2022). Many of the most effective initiatives to ensure child rights were a result of collaborative efforts between grass-roots organizations and municipalities (Gill, 2015; Places for People, 2018; Mayor of London, 2019; Dialogue and Development Commission of Delhi, 2022). Cities have used UNCRC principles, implementation frameworks, tools and expertise from UNICEF and other international organizations to develop child-friendly city plans (City of North Vancouver, no date; NIUA, 2018; Mayor of London, 2019). However, the duty-holders responsible for upholding child rights in changing environments span the full range of agents and actors across society, and failure to meet obligations at any level may result in violations of children’s human rights (Fig. 2).

## 4 Discussion

This article provides a high-level, descriptive overview of the status of children’s rights related to major characteristics of urban environments in Accra, Delhi, London and Vancouver. We found evidence for unequal exposures to risk and opportunity across contexts, with ongoing violation of child rights related to car-based transportation systems, and emerging risks from imbalances between urban services and infrastructure and population increases in the context of climate change. Our findings suggest that fundamental changes in “patterns of production, consumption and transportation” (Landrigan et al., 2019) and collective action from duty-holders at all levels of society are urgently required to address violations of children’s human rights arising from characteristics of urban environments.

**Fig. 3:**
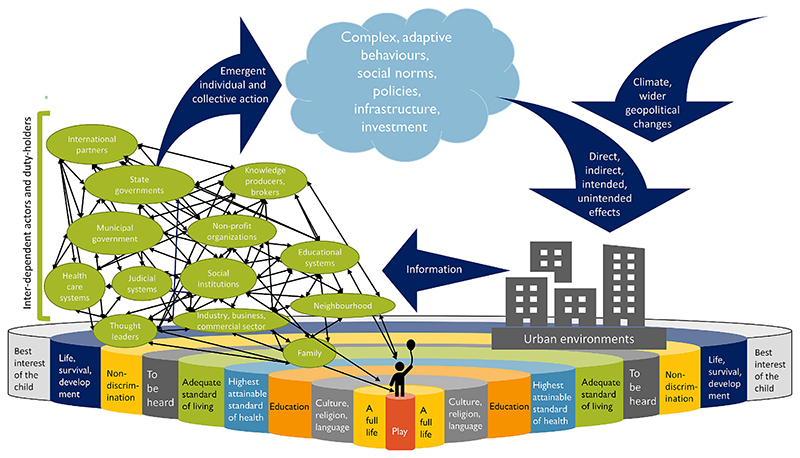
Child rights to healthy urban environments as foundational, unifying principles for motivating, informing and evaluating multi-sectoral action towards sustainable, healthy cities (adapted from Graham, 2013).

Near-universal ratification of the UNCRC by member states is an immense achievement, reflecting the force and relevance of its principles across diverse populations. However, many experts consider the UNCRC to be incompletely theorized from a legal standpoint, and prone to subjective interpretations that are likely to vary across contexts and cultures, while clear allocation of responsibilities for ensuring rights is absent (O’Neill, 2005; Quennerstedt, 2013). This “flexibility and vagueness”, enabling broad acceptance across diverse contexts, is seen by some as limiting the power of the Convention to achieve real change (Quennerstedt et al., 2018, p. 41). States may derive public and international approbation by becoming parties to the Convention while avoiding enforceable commitments. They may aspire to, but lack the economic resources, political will or stability necessary to achieve realization of human rights for children in practice. Even when legal and policy action is taken, political systems and economies often determine the extent to which rights related to resources, environments and services can be ensured by governments (O’Neill, 2005). The emergent properties of cities arise from the interactions of many actors at all levels of society, across time and space. Rather than conceptualizing urban change as primarily hierarchical, complex, top-down organization, Johnson (2001), speaks of cities’ ability to “… self-organize out of millions of individual decisions, a global order built out of local interactions.” Though adopted by State governments to inform legislation and policy, the UNCRC is meaningful and compelling across multiple sectors. Reflecting deeply-held values that resonate with people at all levels of human society, UNCRC articles provide a simple, powerful framework around which cities may dynamically “self-organize” to become sustainable, healthy and resilient (Fig. 3). Such self-organization requires that the UNCRC principles be internalized as core values, expectations and obligations at all levels of society. The capacity of human rights to act as “elements of emancipation” and change depend on their transformation from aspirational or legal frameworks to a collective, “emergent common sense”, that is manifested within social practices (Hunt, 1990, p. 325). The original drafters of the UNCRC articles believed in the power of words that express ideals to create a common vision. Perhaps the most powerful, yet under-utilized potential of the UNCRC lies in its power to motivate collective action when people internalize, enact and expect that these ideals become realities (Fig. 3).

Our findings, focused on specific child rights related to urban environments largely align with previous reports on indicators of child well-being and the status of child rights (NIUA, 2018; Sanderson et al., 2020). A recent report on the status of urban children in India found similar issues: inequality in exposure to traffic, water, sanitation, air pollution and heat risk and unequal access to adequate housing, greenspace and other urban amenities (NIUA, 2018). In *Cities for Children: Children’s Rights, Poverty and Urban Management*, Bartlett et al. (2021) focused on the role of local governments, also identifying city leaders as key actors in upholding child rights and calling for awareness of children and their rights at all levels of society. Our analysis adds to existing knowledge by specifically focusing on the impact of modifiable urban environments on child rights and highlighting a convergence of issues from widely diverse urban settings. Recognizing the critical roles of State and municipal governments, our results emphasize the roles of duty-holders “in small places, close to home” and the need for child rights principles to be known and applied across sectors (Roosevelt, 1999).

A major strength of this case series stems from the collaboration of multi-disciplinary co-authors with subject-area expertise and deep local knowledge of each city’s cultural, policy and physical context. Our findings were strengthened by independent examination of evidence by multiple authors to validate major themes. Cross-case analysis enabled us to the identify patterns across cities that informed higher-level conceptual conclusions (Yin, 2018). We also recognize some important limitations in this work. It provides a high-level overview rather than an exhaustive report of urban environmental factors influencing eleven child rights articles. Because of the need to limit the already broad scope of our investigation, we focused on common exposures related to outdoor urban environments and did not examine toxic (e.g. micro-plastics, pesticides, heavy metals) or detailed aspects of indoor environments that likely impact urban child well-being. Our data sources were limited to those publicly available, with evidence sometimes specific to certain locations or populations within the city. Though we highlighted child and parent perspectives from documentary evidence, interviews with key informants may have provided additional insights. Despite these limitations, this multiple-case study provides an overview of child rights in the study cities and provides cross-case insights on major issues and the potential for a child rights framework to motivate change. Future work is needed to inform effective promotion of UNCRC principles across multiple sectors, actors and ages to fully develop the potential for these principles to inform individual and collective action for healthy, equitable and sustainable urban environments.

## 5 Conclusion

Evidence from four global cities shows that across geographically, culturally and socio-economically diverse contexts, many violations of children’s human rights arise from existing car-based transportation systems. We also identified emerging risks to child health and wellbeing resulting from imbalances between urban services/infrastructure and rapid population increases in the context of climate change that disproportionately impact children from low income and marginalized groups across contexts. Our findings emphasize the urgent need for reimagining existing urban systems and infrastructure that have become incompatible with the deeply and nearly universally held principles of children’s human rights. A human rights framework that centers children’s needs may be the most compelling, effective, unifying foundation for action across sectors to address challenges affecting all members of society. Meaningful commitments to actualization of UNCRC articles in urban environments can benefit entire societies, through sustainable development and urban design that considers children as humans in the present moment and imagines their bright futures.

## Figures and Tables

**Table 1:** Multi-case study search strategy.

UNCRC Article	Broad search criteria	Potential urban characteristics
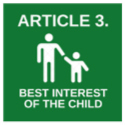	Evidence for urban environmental characteristics that support or undermine the best interest of the child, with a focus on child health, development and well-being.	Greenspace (Vanaken and Danckaerts, 2018; Ye et al., 2022) Space for play, physical activity, social connection (Gemmell et al., 2022)
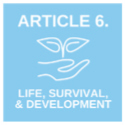	Evidence for features of urban environments that support the right to life, survival and development.	Active travel environment (Rothman et al., 2021, 2022) Noise (Stansfeld and Clark, 2015) Air quality (Holm et al., 2021)
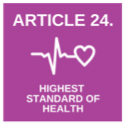	Evidence for urban environmental impacts on children’s ability to realize their highest attainable standard of health.	Traffic environment (Quistberg et al., 2022; Rothman et al., 2022) Housing (Qiu and Zhu, 2017; Nasim, 2022) Water, Sanitation (Fink et al., 2011; Ngure et al., 2014)
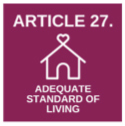	Evidence for urban environmental influences on children’s attainment of a standard of living adequate for physical, mental, spiritual, moral and social development.	Natural space (Lee et al., 2012)
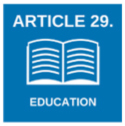	Evidence for impacts of urban environments on children’s right to education that prepares them for a responsible life in a free society, and develops respect for the natural environment.	Natural spaces, biodiversity (Salmón, 2000)
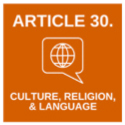	Evidence for how urban environments influence child rights to culture, religion and language.	
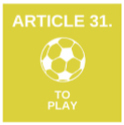	Evidence for how urban environments influence children’s access to play.	Space for play (Derose et al., 2018; Gemmell et al., 2022) Greenspace (Grigsby-Toussaint et al., 2011) Traffic environment (Flowers et al., 2019) Social space (Adjei-Boadi et al., 2022; Gemmell et al., 2022)
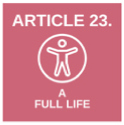	Evidence for urban environmental influences on children’s opportunities in conditions that promote dignity, self-reliance, active participation.	Mobility (Morales et al., 2018) Accessible space for play (Shields et al., 2012)
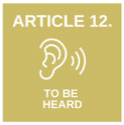	Evidence of serious consideration of child perspectives regarding urban environments.	Child perspectives in urban planning processes (Feldner et al., 2019)
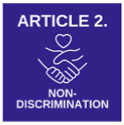	Inequalities in exposure to health enabling or harmful features within urban environments based on racial, ethnic, caste, gender, socioeconomic, ability or other differences.	Equity issues related to urban environmental exposures (Browning et al., 2022)
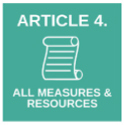	Evidence for application of all measures and resources to issues impacting child rights related to urban environments.	Extent to which identified issues are addressed, prioritized in the context of each city (Payne, 2017)

**Table 2:** Characteristics of study cities.

Name	Study Definition	Area (km^2^)	Percent 19 years and under (%)^c^	Population totals (n)
Accra^a^	Greater Accra Metropolitan Area (GAMA)	1585	38	5,455,692
Delhi^b^	National Capital Territory (NCT) of Delhi	1484	37	20,965,000
London^a^	Greater London (32 boroughs and City of London)	1572	25	9,002,488
Vancouver^a^	Metro Vancouver (21 municipalities, one electoral area, one Treaty First Nation)	2879	19	2,642,825

a Based on census data, 2021 (Ghana Statistical Service, 2021; Nomis Office for National Statistics, 2021; Statistics Canada, 2022a).

b NCT of Delhi is surrounded by the National Capital Region (NCR), also commonly called “Delhi” that consists of urban, suburban and rural areas (National Capital Region Planning Board, 2018). Data presented here is for the NCT of Delhi only. Demographic data based on population projections for 2020 from the Government of India. (Government of India).

c Percent population under 20 years is given to enable comparisons across cities, as age structure was only available in 5-year categories for some cities.

**Table 3:** Characteristics of urban environments and their impacts on child rights across study cities.

Urban environmental characteristics	Accra, Ghana	Delhi, India	London, UK	Vancouver, Canada	UNCRC rights impacted
Greenspace	Inequality in access based on socio-economic status (Adjei-Boadi et al., 2022)	Inequality in access based on socio-economic status. (Mitchell et al., 2021)	Inaccessible to some children due to social safety concerns, family resources and time. (Allport et al., 2019)	Inequality in neighbourhood greenness based on socioeconomics (Quinton et al., 2022) Most children live near a park but may not be able to access it due to unsafe routes, social safety risks, risk-averse parenting norms. (Gerlach et al., 2019) Play Street and School Street initiatives have been launched to allow children to play on roadway for short periods. (TransLink, 2022)	Articles 2, 3, 4, 6, 12, 23, 24, 27, 30, 31
Natural space	Poor maintenance of public play and greenspaces (Mmofra Foundation, 2020; Adjei-Boadi et al., 2022)	*Raahgiri* Day is a car-free citizen initiative that reclaims streets as public spaces for all by temporary closure of roads (Dialogue and Development Commission of Delhi, 2022).	Lack of adequate greenspace and play space for school children in Central London (Shoari et al., 2021).		
Playable space	Ghanaian non-profit organizations advocate for provision of green and play space and inclusion of children in planning of such spaces (Mmofra Foundation, 2020).	School playgrounds may be appropriated for adult purposes (Singh, 2018).	Play Streets provide children with opportunity for social connection, physical activity and play in some boroughs (Gill, 2015).		
Adequate, safe housing Secure housing tenure	Inadequate housing stock, unsafe housing poses risks due to flooding, (Amoako and Boamah, 2015) infectious diseases, extreme heat (Wilby et al., 2021), fire (Frost et al., 1998) and social safety. Residents in informal settlements lack legal tenure and may be subject to forced eviction (Afenah, 2009).	Inadequate housing stock, unsafe housing poses risks due to flooding, infectious disease, extreme heat, fires, social safety. Residents in informal settlements lack legal tenure and may be subject to forced eviction (Manish, 2022).	Over 75,000 children are homeless or living in temporary accommodations due to shortage of social rental housing and increasing housing costs. (Firth, 2022; Munro et al., 2022) Overcrowding is high in social housing (Munro et al., 2022). Inadequate housing quality, dampness, cold and mould increase child respiratory illnesses and adversely affect mental health (Munro et al., 2022).	High housing costs (Metro Vancouver Regional Government, 2015; Cuthbert, 2022; Meissner, 2022) contribute to higher family stress, fewer opportunities and, resources to support child development, lack of secure tenure, and higher residential mobility, which may negatively influence development of sense of community and social capital (Gerlach et al., 2019; Todd, 2021).	Articles 2,3, 4, 6, 23, 24, 27
Traffic	Road traffic deaths accounted for 61.5% of injury-related deaths at an Accra teaching hospital, of these, 50% occurred in pedestrians (Blankson et al., 2019). Insufficient pedestrian infrastructure or deteriorated paths, obstructions are barriers to walking. Informal activities (e. g. markets, hawkers) may obstruct walkways, cars may compete with pedestrians for walkways space (Amegah, 2022).	Pedestrians at highest risk for traffic-related deaths, young males nearly three times more likely to be killed by traffic than females (Traffic Management Division, 2021). Inadequate pedestrian, cycling infrastructure and traffic calming around schools (Tarun et al., 2017).	Increasing socio-economic inequality in child pedestrian safety over time (2010–2020), with higher injury among more disadvantaged groups (Shoari et al., 2023). Safe travel to school is a concern as high traffic-related injuries in children were seen in areas with more schools (Shoari et al., 2023).	Traffic risks limit children’s freedom to access local environments. Parental concerns limit child mobility and outdoor play (Vlaar et al., 2019). Traffic congestion at school drop-off and pick-up poses risks to children. New initiatives targeting changes in transportation mode to school to reduce the number of vehicles and increase active travel to school (TransLink, 2022).	Articles 2, 3, 4, 6, 12, 23, 24, 27, 29, 30, 31
Clean water, sanitation	Many low-income families experience insecure access to water (Kangmennaang et al., 2020). Estimated 80% of households drink sachet water due to lack of access to piped water or perceptions of poor quality of piped water (Moulds et al., 2022). In a study of 5 informal settlements, only <32% had access to improved sanitation facilities (Kangmennaang et al., 2020).	83% of Delhi households have access to piped water supply, water tankers supply areas without piped water, but some still rely on untreated water sources. Groundwater depletion is a concern due to high consumption and increasing impervious surface area. Persistent inequality in water access (Biswas and Gangwar, 2021; Kumar et al., 2021).	Water delivery is administrated by private companies who monitor quality. Residents can request testing (City of London, 2023). Environment agency enforces quality standards (GOV. UK, 2023).	Routine testing of daycare and school tap water is carried out in Vancouver (Wachtel et al., 2017), tap water in homes is not routinely tested (Wells et al., 2019).	Articles 2, 3, 4, 6, 23, 24, 27
Active travel, mobility	Children generally play in informal spaces close to home. However, traffic risks and lack of pedestrian infrastructure pose serious risks that may lead parents to limit children’s independent mobility (Adjei-Boadi et al., 2022).	Children cannot ride bicycles on the street or walk to school unsupervised in large metropolises like Delhi due lack of safe pedestrian and cycling infrastructure (Tarun et al., 2017). Despite laws to promote accessibility, children with disabilities are excluded from many urban spaces due to lack of accessibility infrastructure (NIUA, 2018).	Initiatives implemented to increase safety for children’s active travel in some boroughs. Actions include reduced speed limits, improved pedestrian crossings, segregated cycle lanes, pocket parks, tree planting, bike hangers, removal of parking spaces.	Free public transit for children under 12, walking school buses, cycling classes and other initiatives seek to support children’s active transportation (TransLink, 2022; Society for Children and Youth of BC, 2023). Lower-income youth (13–18) may lack access to transportation (Lazaruk, 2021). Safe pedestrian and cycling infrastructure exists in higher density areas (Winters et al., 2022), however, peripheral areas may lack infrastructure for safe, active travel (Metro Vancouver Regional Planning, 2023).	Articles 3, 4, 6, 12, 23, 24, 27, 29, 30, 31
Air quality	PM_2.5_ exposure 7.2 times higher, on average, than World Health Organization (WHO) guidelines, with higher exposures among girls (Arku et al., 2015).	Poor air quality experienced by all children in Delhi with local PM_2.5_ levels up to 400 μg/m^3^ (WHO air quality guideline for PM_2.5_ is 5 μg/m^3^) (Selokar et al., 2020; World Health Organization, 2021)	Children attending schools in Central London have a high probability (>90%) of exposure to NO_2_ levels exceeding WHO guidelines (40ug/m^3^) (Shoari et al., 2022).	Higher exposure to NO_2_ and PM_2.5_ at schools in lower income neighbourhoods where school is more likely to be located close to a major road (Amram et al., 2011). Seasonal wildfire smoke exposure is increasingly frequent and poses serious health risks to children (Holm et al., 2021).	Article 2, 3, 4, 6, 23, 24, 27, 31
Noise	Noise levels for most children consistently above WHO guidelines, most attributable to road traffic noise with higher exposures in low-income neighbourhoods (Clark et al., 2021)	Noise levels exceed WHO recommended guidelines across Delhi, with most attributable to road traffic noise. (Chauhan et al., 2023)	Modelled noise exposure across London showed that 19% of the population was exposed to daytime noise above WHO guidelines, while 100% were exposed to nighttime noise above guidelines (Gulliver et al., 2015).	Higher noise exposure in schools in lower-income neighbourhoods which tend to be closer to major roads (Amram et al., 2011).	Articles 2, 3, 4, 6, 23, 24, 27, 30, 31
Climate change-related risks	Flooding risk, especially for informal settlements on flood plains (Amoako and Boamah, 2015). Extreme heat events, exacerbated by neighbourhood and housing characteristics (Wilby et al., 2021).	Flooding risk, especially for informal settlements on flood plains (Tomar et al., 2021). Extreme heat risks, particularly for children in low income and marginalized groups (Mitchell et al., 2021).	Extreme heat events (Giordano, 2022), urban flood risk due to increasing population, increasing impervious surfaces and pressures on urban drainage systems (Greater London Authority, 2018b).	Increasing frequent and severe seasonal wild-fire smoke exposures are expected, posing serious child health risks (Matz et al., 2020). Health risks due to increasingly frequent extreme heat events (Henderson et al., 2022).	Articles 2, 3, 4, 6, 12, 23, 24, 27, 29, 30, 31

## Data Availability

All data used in this case series is publicly available. Additional materials generated during within and cross-city analyses will be made available upon request from the corresponding author.
